# Proteinaceous Lung With COVID-19: The Mimicker

**DOI:** 10.7759/cureus.18144

**Published:** 2021-09-20

**Authors:** Surbhi Surbhi, Yudhyavir Singh, Kapil Dev Soni, Anjan Trikha

**Affiliations:** 1 Anaesthesiology, Critical Care and Pain Medicine, All India Institute of Medical Sciences, New Delhi, New Delhi, IND; 2 Anesthesia and Critical Care, All India Institute of Medical Sciences, New Delhi, New Delhi, IND; 3 Critical Care, JPN Apex Trauma Centre, All India Institute of Medical Sciences, New Delhi, New Delhi, IND; 4 Anaesthesiology, Pain Medicine and Critical Care, All India Institute of Medical Sciences, New Delhi, New Delhi, IND; 5 Anaesthesiology, All India Institute of Medical Sciences, New Delhi, New Delhi, IND

**Keywords:** ild, crazy waving pattern, ground glass opacity, covid-19, pap

## Abstract

Pulmonary alveolar proteinosis (PAP) is a syndrome, in which surfactants get deposited slowly in alveoli, blocking the airway exchange. PAP severity also varies from mild to severe, presenting with dyspnea, cough, hemoptysis with or without fever. The radiological findings are ground-glass opacities along with septal thickening (Crazy Paving), consolidations, and less commonly air bronchograms. COVID-19 is a viral infection caused by SARS COV2 primarily affecting the lungs and causing atypical viral pneumonia. The clinical picture of the disease varies from a milder form of fever, dry cough with or without expectoration, to severe disease-causing respiratory distress, pneumonia, acute respiratory distress syndrome (ARDS), and even death. Radiologically, the findings of COVID-19 are similar to PAP. So, PAP mimics the COVID-19, posing a differential challenge, though our patient was a known case of PAP. Therefore, for proper management of the disease, it is important to differentiate it from other pathologies. In this case report, we describe a patient who was a known case of autoimmune pulmonary alveolar proteinosis. She presented with acute exacerbation in the emergency department and tested positive for COVID-19. We followed a systematic approach consisting of clinical, laboratory, radiologic parameters to differentiate the cause of this exacerbation.

## Introduction

Pulmonary alveolar proteinosis (PAP) is a rare, non-infectious disease, characterized by the deposition of amorphous lipoproteins in the alveoli that impairs gas exchange, causing hypoxia. Autoimmune PAP accounts for 90% of cases. The incidence of PAP is 0.2 cases per million and commonly involve male [1,2]. Herein, we describe a female patient of PAP in her early 1930s, who presented with acute exacerbation and was diagnosed with COVID-19 simultaneously.

## Case presentation

A 34-year-old lady, known case of autoimmune “PAP,” presented to the hospital with a history of low-grade fever, breathing difficulty, and a dry cough from the last four days in the emergency department (ED). She was diagnosed with PAP two years back and had received one total lung lavage (TLL) therapy for the same. She remained asymptomatic for ten months and thereafter, with symptoms appearing again, she was treated with granulocyte-macrophage colony-stimulating factor (GM-CSF) therapy by the inhalation route, 12-doses over for five to six months. She remained asymptomatic until recently presented with breathing difficulty in ED. She was conscious, her vitals were stable and oxygen saturation on the face mask (10 liters/min) was 96%. Following admission, she was to undergo another session of TLL when during pre-procedure workup she tested positive for COVID-19. The procedure was postponed due to the high chance of infectivity via aerosol generation, and she was shifted to the COVID-19 intensive care unit (ICU) for further management.

**Figure 1:**
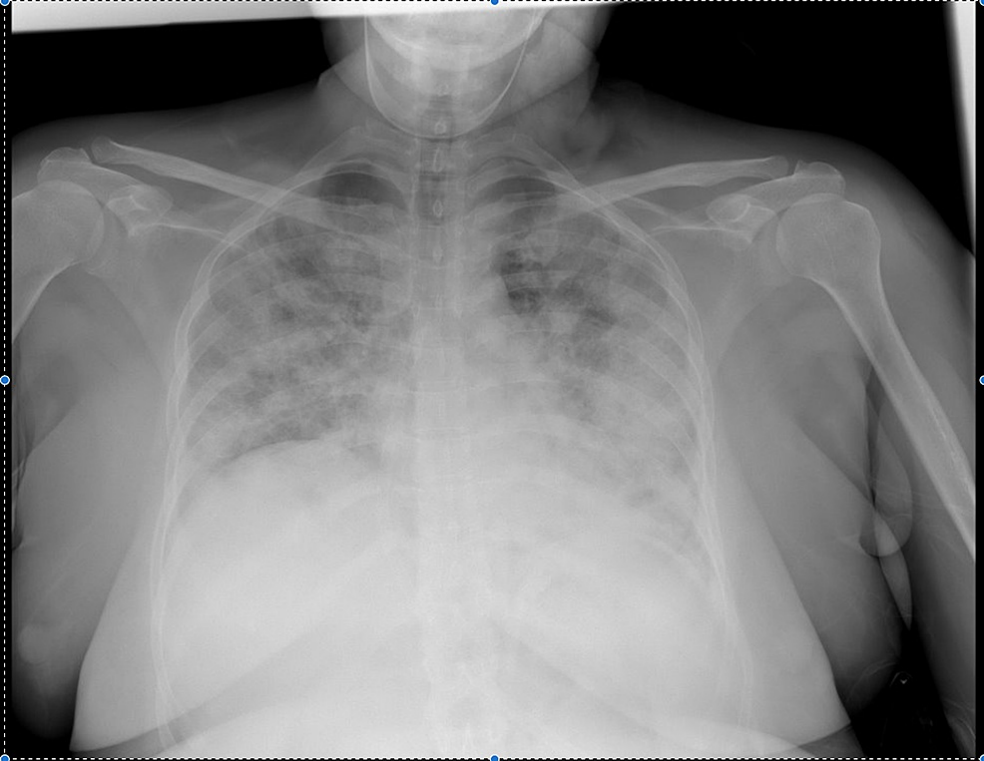
Chest X-ray PA view (bilateral diffuse opacities).

**Figure 2:**
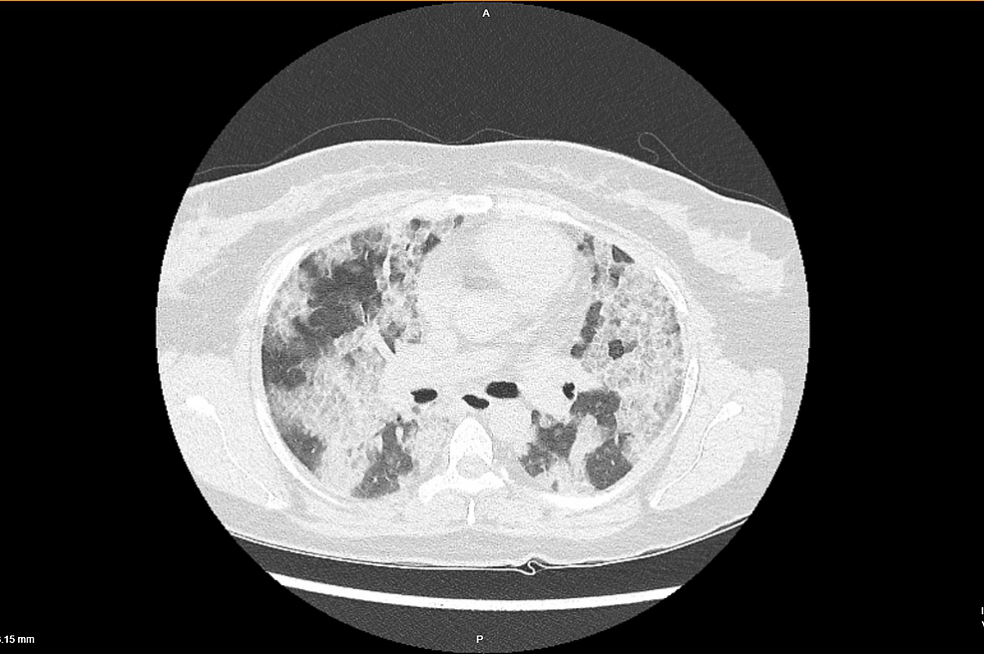
HRCT chest transverse section (Crazy-Paving pattern and ground-glass opacities). HRCT: high-resolution computed tomography.

In the ICU, she was put on a face mask (10 liters/min) with a target saturation of 95%. She was treated with an antimicrobial, steroids (low dose methylprednisolone), and antipyretics. Her blood investigations revealed lymphopenia, though the leukocyte count was normal and this lymphopenia persisted throughout the hospital stay (Table 1). Rest blood parameters were normal including the inflammatory markers (Table 1). Chest X-ray showed bilateral infiltrates suggestive of severe ARDS (Figure 1). HRCT scan of the lung shows multifocal bilateral infiltrates and ground-glass opacities with consolidations having basal prominence (Figure 2). The patient responded to the treatment with a decrease in oxygen requirement and was transferred back to the non-covid facilities after the reverse transcription-polymerase chain reaction (RT-PCR) report came negative for COVID-19. Her stay in COVID-19 ICU was eight days. She underwent TLL therapy and was finally discharged.

**Table 1:** Laboratory data. SGOT: serum glutamic-oxaloacetic transaminase, SGPT: serum glutamic pyruvic transaminase, PT: prothrombin time, INR: internationalized normal ratio, APTT: activated partial thromboplastin time, IL6: interleukin-6, CRP: C-reactive protein, LDH: lactate dehydrogenase.

Variable	Day 1	Day 3	Day 7	Discharge	Normal range
Hemoglobin (g/dL)	11.3	11.2	11	12	12-15
Total leucocyte count (10^3^ /µL)	7.1	7.8	10.9	11.0	(10–26)
Neutrophils (%)	63	89	82	89	40–75
Lymphocytes (%)	27	10	15	10	20–40
Platelets (10^3^/µL)	207	188	217	282	150–400
Hematocrit (%)	39	37.7	38	40.3	36–46
Total bilirubin (mg/dL)	0.8	0.7	0.5	0.6	0.3–1
Direct bilirubin (mg/dL)	0.3	0.2	0.1	0.1	0–0.2
SGOT/SGPT (units/L)	30/18	43/19	31/24	24/19	5–40
Total protein (g/dL)	7	6.7	6.3	6.4	6–8.7
Albumin (g/dL)	3.9	3.8	3.9	3.7	4–5.5
PT (seconds)	14.9	13.9	14.7	15	10.7–15.3
INR	1.11	1.03	1.09	1.12	<1.1
APTT (seconds)	35.8	30.8	31	35	30–40
Urea (mg/dL)	11	19	23	30	10–50
Creatinine (mg/dL)	0.5	0.5	0.4	0.4	0.5–1.2
Sodium (mmol/L)	139	136	137	137	130–149
Potassium (mmol/L)	4.2	4	3.8	4.2	3.5–5
Calcium (mg/dL)	9.4	8.7	8.8	9.6	8.1–10.4
Phosphate (mg/dL)	3.1	5	4.6	4.8	2.5–4.5
Magnesium (mg/dL)	2.09		2.26		1.46–2.68
IL6 (pg/mL)	5.3	9.01	2.1	6.64	5–15
Ferritin (ng/mL)	19.5	22	22.2	34.1	12–300
CRP (mg/L)		3.42	0.3	2.7	<5
D dimer (ng/mL)	129.89				0–255
Procalcitonin (ng/mL)	0.03	0.02	0.01	0.01	<0.1
LDH (U/L)	338	353	526	427	140–280

## Discussion

PAP is a rare type of diffuse interstitial lung disease (ILD). It is characterized by the deposition of surfactant protein within the alveoli; affecting gaseous exchange, because of failure of this accumulated protein to get clear, causing hypoxia [1]. While PAP is a non-infective ILD whereas COVID-19 is a viral infection caused by SARS-CoV-2 primarily causing pneumonia. The treatment of choice for PAP is TLL while for COVID-19, treatment is mainly supportive and symptomatic, to date.

The clinical features and radiological findings of PAP resemble those observed in COVID-19. However, other lung pathologies which mimic COVID-19 include pulmonary edema, organizing pneumonia, aspiration pneumonia, pulmonary hemorrhage, pulmonary neoplasms, sarcoidosis, pulmonary infarction, and ILD [3]. PAP commonly presents with persistent dry cough sometimes associated with scanty sputum, progressive dyspnea, fatigue, malaise, intermittent low-grade fever, and pleuritic chest pain [1,2]. Most of the symptoms are nonspecific and might be confused with that of COVID-19 pneumonia, but symptoms in ILD are mostly chronic whereas in COVID-19 is acute. In our case, the patient presented with worsening dyspnea for the past month, dry cough with occasional expectoration, and fever of recent onset which could have been either acute exacerbation or new infection of COVID 19.

Chest radiography of PAP shows bilateral diffuse alveolar opacities without air bronchogram with the perihilar and basal distribution. The most frequent computed tomography findings of PAP are diffuse ground-glass opacities, intralobular thickening, and parenchymal consolidation. This pattern is called “Crazy Paving,” which is specific for PAP but not pathognomonic. Opacities have a typically geographic distribution, with the juxtaposition of healthy and sick zones. The zonal distribution is usually not specific; however, lower zone predominance is seen. A chest CT scan is a major tool for the diagnosis of PAP [1].

Similar CT findings like diffuse ground-glass opacities superimposed with interlobular and intralobular septal thickening were recently reported in COVID-19 pneumonia. Other CT findings are peripheral opacities with consolidations, and/or septal thickening, less commonly air bronchograms, CT halo sign, and reverse halo sign [3,4]. All these radiological findings have been initially described in PAP. COVID-19 patients may show similar radiological findings to those of ILD because of pulmonary inflammation, alveolar edema; fibrin, hyaline membrane, and cell infiltrations; and associated interstitial changes associated with them. Comparing with our patient, similar findings in chest radiograph (Figure 1), as well as CT chest, were present (Figure 2). In COVID-19, chest CT has been commonly used for diagnosing as well as quantifying the severity of lung involvement [4]. 

Owing to the similarity in the clinical presentation and radiology of both, any delay in the differential diagnosis of these diseases with completely different treatment, may fail to isolate the COVID-19 case or to treat an ILD (PAP) properly to prevent progression or even mortality. However, here our patient was already diagnosed with PAP, so no diagnostic challenge. A detailed history with clinical symptoms assessment, along with laboratory parameters and radiological evaluation should be done to find out whether this exacerbation was due to PAP or a mild form of COVID-19, or it was primarily a severe COVID 19 ARDS picture.

The PAP is diagnosed with periodic acid-Schiff staining of bronchoalveolar lavage fluid (turn positive) and its cytological examination (shows increased cellularity) [2]. The positive RT-PCR test for COVID-19 confirms the diagnosis. However, to track further courses, it is essential to monitor laboratory parameters including inflammatory serum biomarkers. In COVID-19, lymphocytopenia and neutrophilia is a prognostic indicator and is correlated with the severity of disease requiring ICU care, ARDS, and increased mortality [5]. Acute phase reactants due to inflammation may be normal or moderately elevated in ILD while it is mild to markedly raised in COVID-19 [6]. Similarly, our patient also exhibited lymphopenia with a higher percentage of neutrophils, but leucocyte counts and acute phase reactants were within the normal range. IL-6 level is increased in COVID-19 patients and a high baseline value is correlated with severe disease and increased mortality [7]. Furthermore, C-RP and IL-6 are used to monitor therapeutic response. However, the relevance of ferritin levels in COVID-19 patients is equivocal and yet to be ascertained [8]. Elevated D-dimer levels are seen in COVID-19 patients and a level >2.0 mcg/ml on admission predicts mortality [9]. Our patient was a diagnosed case of PAP, and she had responded to previous TLL, GM-CSF therapy, but this time co-infection with COVID-19 made it uncertain how the disease will progress. We found that mild COVID-19 did not influence the patient outcome.

## Conclusions

PAP mimics clinically and radiologically with COVID-19 but, both are different diseases and can complicate and can cause a diagnostic dilemma when they co-exist. The pathophysiology and management of PAP and COVID-19 are different; the PAP being a non-infective ILD whereas COVID-19 is an infective viral disease caused by SARS-CoV-2 primarily causing pneumonia. Any delay in diagnosing these two diseases can lead to poor patient outcomes by failing to isolate a COVID-19 case.
